# Supplementary material for: Antibiofilm Activity and Biocompatibility of Temporin-SHa: A Promising Antimicrobial Peptide for Control of Fluconazole-Resistant Candida albicans
Source: Microorganisms. 2024 Jan 4;12(1):99. doi: 10.3390/microorganisms12010099 (PMC10818419; doi:10.3390/microorganisms12010099)
Supplement: Supplementary file 1 [file microorganisms-12-00099-s001.zip › microorganisms-2690690-supplementary.pdf]

SUPPLEMENTARY FILE:

The significant viability reduction (≥50%) was observed after longitudinal exposure of CaS planktonic culture to Fluconazole (Figure S1-a) compared to the control ($p \leq 0.05$) after 6 and 24h, at concentrations of 256 µg/mL (6h: 57%; 24h: 58%), 512 µg/mL (6: 54%; 24h: 59%), and 1024 µg/mL (6h: 62%; 24h: 68%). Regarding the results of CaR after the longitudinal exposure to Fluconazole (Figure S1-b), significant reduction (≥50%) were observed compared to the control group ($p \leq 0.05$) after 6 and 24h at concentrations of 256 µg/mL (6h: 54%; 24h: 58%), 512 µg/mL (6h: 56%; 24h: 59%) and 1024 µg/mL (6h: 62%; 24h: 65%).

a) b)

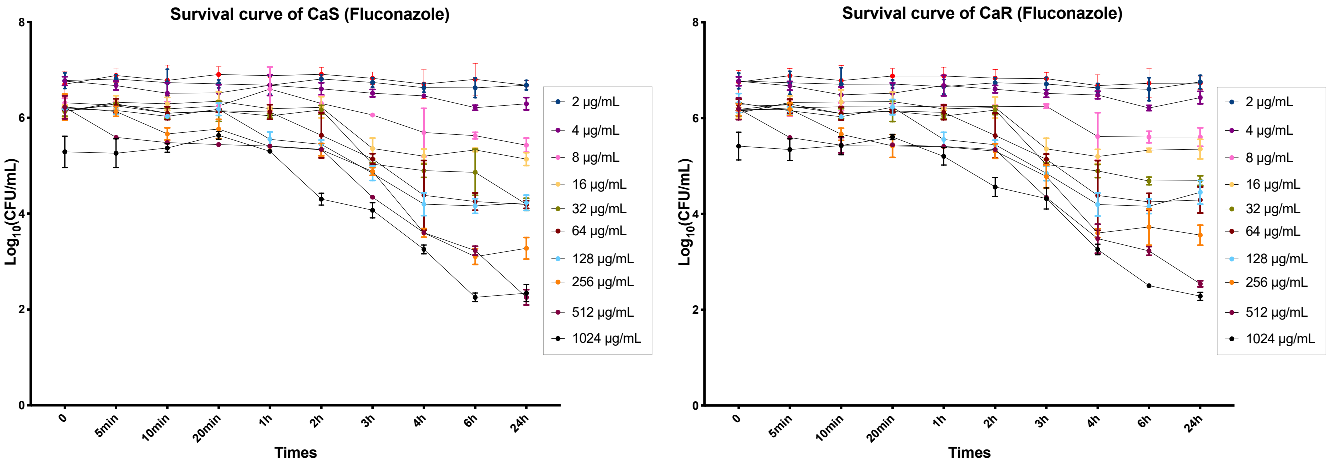

Figure S1. Mean and 95% confidence interval of survival in $\log_{10}$ of CaS (a) and CaR (b) suspensions after longitudinal exposure to predetermined concentrations (2, 4, 8, 16, 32, 64, 128, 512 and 1024 µg/mL) of Fluconazole at predetermined times (0; 5 min; 10 min; 20 min; 30 min; 1 hour; 2 hours; 3h; 4h; 6h and 24h). Points: data averages. Error bars: minimum and maximum values. The non-intersection of the error bars denotes a statistical difference according to the 95% confidence interval (n=12/group; $p<0.05$).

For treatment with a standardized drug, Fluconazole, the concentration capable of a reduction of ≥50% MFC of CaS (Figure S2-a) and CaR (Figure S2-b) viable colonies was ≥256 µg/mL. The highest CaS and CaR viable colony reductions were found at 1024 µg/mL in 66% and 60% (approximately 5 $\log_{10}$) reduction for CaS and CaR, respectively, with this being statistically different from the control group ($p \leq 0.05$).

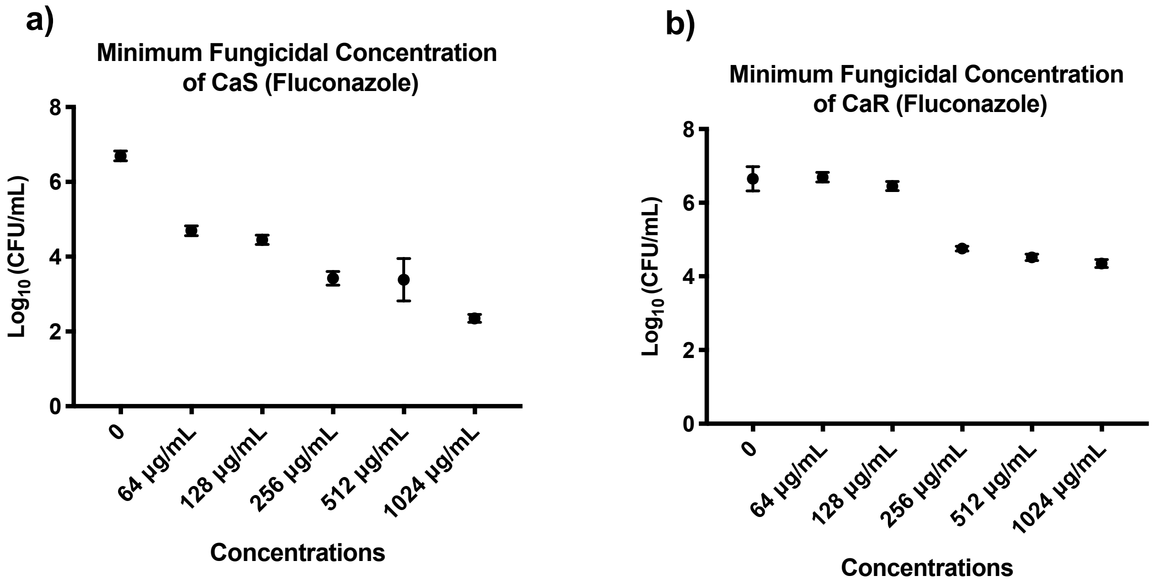

Figure S2. Mean and 95% confidence interval of the MFC (log_{10}) of CaS (a) and CaR (b) suspensions after exposure to Fluconazole at 64, 128, 256, 512 and 1024 µg/mL. Points: data means. Error bars: minimum and maximum values. The non-intersection of the error bars denotes a difference according to the 95% confidence interval (n=12/group; $p<0.05$).
